# Nanoscale-enabled cross-scale chemical sensing for underwater monitoring and beyond

**DOI:** 10.3762/bjnano.17.103

**Published:** 2026-10-30

**Authors:** Tatsuhiro Fukuba, Nicolas Clement, Henri Vo Van Qui, Yui Sasaki, Koji Toma, Tsuyoshi Minami

**Affiliations:** 1 Research Institute for Marine Technology and Engineering, Marine Engineering Science Center, Marine Observational Technology Research Group, Japan Agency for Marine-Earth Science and Technology, Natsushima-Cho 2-15, Yokosuka, Kanagawa, 237-0061, Japan; 2 Institute of Industrial Science, The University of Tokyo, 4-6-1 Komaba, Meguro-ku, Tokyo, 153-8505, Japan; 3 LIMMS/CNRS-IIS, 4-6-1 Komaba, Meguro-ku, Tokyo, 153-8904, Japan; 4 Research Center for Advanced Science and Technology, The University of Tokyo, Komaba 4-6-1, Meguro-ku, Tokyo, 153-8904, Japan; 5 College of Engineering, Shibaura Institute of Technology, 3-7-5 Toyosu, Koto-ku, Tokyo, 135-8548, Japan

**Keywords:** cross-scale sensing, lab-on-a-chip (LOC), nanoscale sensing, underwater

## Abstract

In aquatic environments, such as oceans, lakes, and rivers, molecular-level chemical reactions and the distribution of chemical species are related to global environmental changes through various biogeochemical processes. Understanding this relationship requires addressing cross-scale challenges at two distinct levels: overcoming interfacial physical constraints, such as Debye screening in high-salinity waters, at the sensing level and bridging local microscale measurements with macroscale environmental dynamics at the observational level. In this paper, we describe sensing technologies that can enable cross-scale sensing in aquatic environments. Here, we focus on nanoscale interfacial sensing and molecular recognition technologies, as well as lab-on-a-chip technologies for *in situ* measurements including power-efficient pumping technologies. Additionally, underwater observation platforms that link the local chemical sensing and global processes are overviewed. Beyond environmental applications, these sensing technologies offer a cross-disciplinary approach that can be extended to analyze biological systems facing similar physicochemical constraints.

## Introduction

Aquatic environments such as oceans, lakes, and rivers are complex systems with interactions among a variety of biogeochemical processes across a wide range of spatial and temporal scales. Distribution of dissolved chemical species and molecular-level biogeochemical processes affect not only the behavior of individuals, communities, and single ecosystems, but also global environmental changes. In ocean environments, from the surface to the deep sea, from the coast to the open ocean, environmental parameters such as temperature, salinity, and concentrations of dissolved chemicals change dynamically across various spatiotemporal scales. These variations propagate across the sediment–water and air–water interfaces and connect the processes within the Earth system. Because these variations have a wide temporal range, it is difficult to capture the whole picture only with a single sensor technology. Moreover, appropriate observation platforms are necessary in addition to sensing devices for practical *in situ* measurements. For example, slight fluctuations in the proton concentration, which affect the equilibrium of the carbonate system in seawater, can be described as acid–base reactions at the molecular level. However, such small changes influence coral calcification rates and plankton community structure and may ultimately affect the marine carbon cycle and global climate change [1]. To measure variations of proton concentration, generally expressed as pH, reliably *in situ*, sensing devices that can capture pH variations with high spatiotemporal resolution are necessary. Furthermore, bridging localized chemical sensing to global monitoring requires a scalable approach that integrates miniaturized sensor devices with autonomous and distributed observation platforms. The international Argo program, which utilizes thousands of profiling floats across the ocean environment, is a representative example of such scalable global observation networks [2]. Recently, this program was expanded to Biogeochemical Argo (BGC-Argo) for the measurement of biogeochemical processes on individual floats with sensors for dissolved oxygen, pH, and nutrients [3]. These advances show the importance of integrating highly miniaturized and reliable chemical sensors and scalable autonomous platforms to realize large-scale environmental monitoring. To address this issue, we propose a cross-scale sensing approach based on molecular-scale sensing, microfluidics to support the sensing, and observation platforms capable of local and global environmental monitoring. Furthermore, this approach may also become a technological foundation for future biological and in vivo sensing applications because aquatic and biological environments share several physicochemical constraints.

## Perspective

### Field-proven sensor technologies

#### Ion-sensitive field-effect transistors (ISFETs)

**Figure 1:**
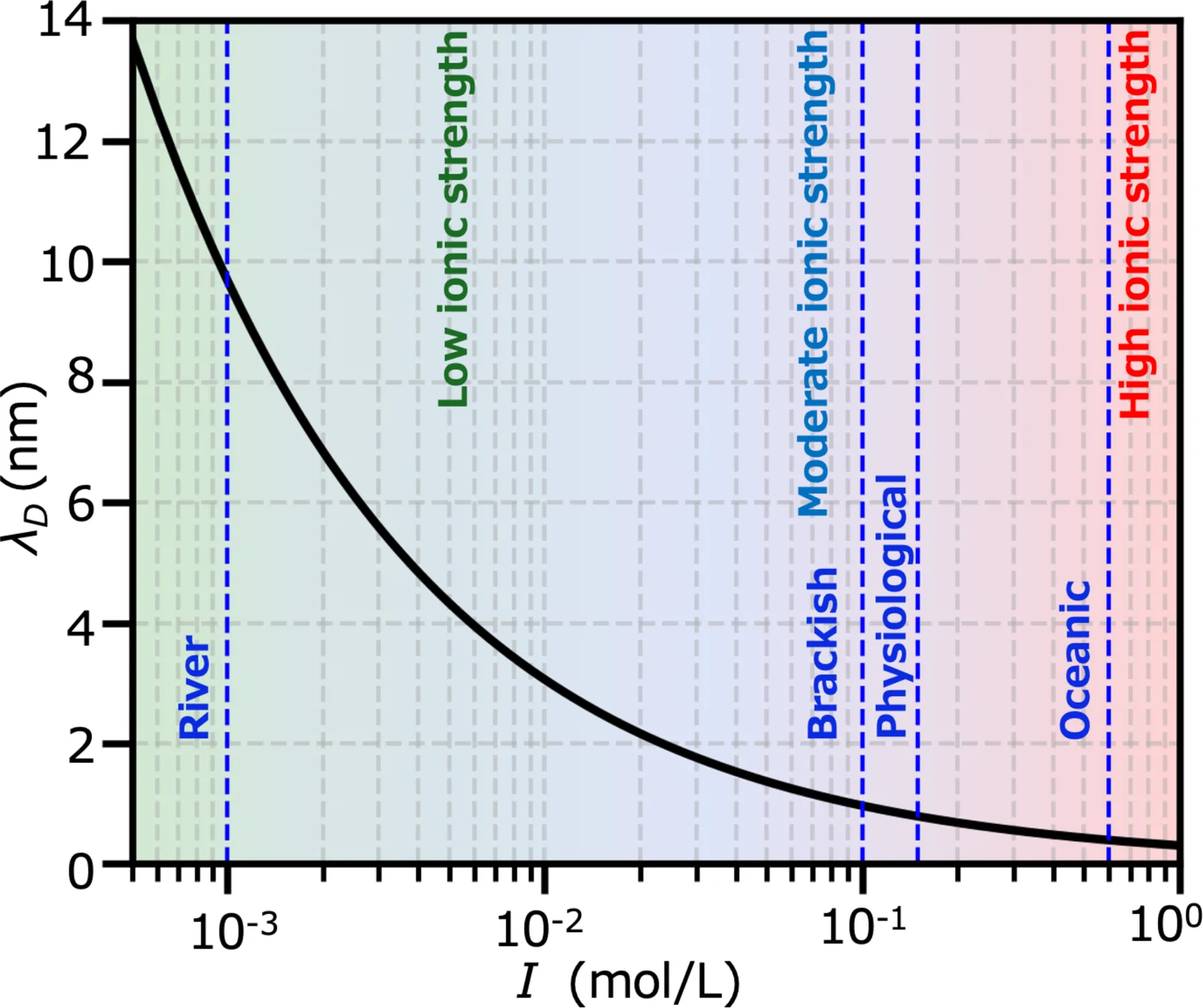
Debye length variation with typical ionic strength across river, brackish, physiological, and oceanic environments.

In aquatic environments, a variety of chemical sensors and biosensors have been developed and utilized for the rapid detection and quantification of dissolved chemical species [4]. For example, glass electrodes are widely used for underwater potentiometric pH measurements [5]. However, glass electrodes have limitations in terms of mechanical robustness and potential for further miniaturization due to their structure. Moreover, periodic maintenance and calibration are required due to the presence of a liquid junction. When such maintenance is not feasible, signal drift results in a decrease in measurement reliability [6]. Alternatively, ion-sensitive field-effect transistors (ISFETs) [7–8] have been noted as robust devices suitable for long-term *in situ* underwater measurements [9–14]. ISFETs are solid-state devices composed of silicon and dielectric materials, and their cavity-free structure provides resistance to hydrostatic pressure changes [9]. The sensitive area of ISFETs used for underwater applications is typically made of dielectric materials such as tantalum pentoxide (Ta_2_O_5_), aluminum oxide (Al_2_O_3_) [9], or silicon nitride (Si_3_N_4_) [14]. The dielectric layer is deposited under high-temperature conditions in clean-room environments, resulting in highly dense, defect-free, and thus mechanically robust and chemically stable crystalline or amorphous structures. Their low output impedance and CMOS compatibility have made ISFETs one of the most established underwater chemical sensors. In particular, pH-ISFETs have been evaluated *in situ*, including deep-sea environments, and their effectiveness has been successfully demonstrated [13]. In subsequent studies, their application has been expanded to the measurement of important carbonate system parameters such as the partial pressure of CO_2_ (*p*CO_2_) [10,14] and total alkalinity [15]. The response of the pH-ISFET arises from protonation and deprotonation reactions occurring at the metal oxide surface [7–8]. Because these reactions occur within the Debye screening length at the solid–liquid interface, the electric potential changes remain detectable even under high-ionic-strength conditions. However, when a functional layer is introduced onto the gate surface for the detection of specific target molecules, Debye screening can limit sensitivity in high-ionic-strength liquids such as seawater. A similar challenge exists in sensor operation within biological systems, which also exhibit high ionic strength comparable to that of seawater [16–17]. The variation in ionic strength from freshwater rivers (<10 mM) to brackish estuaries and marine environments (0.6–0.7 M) [18] makes the design of electrostatic sensing platforms increasingly challenging and complex (Figure 1). Therefore, overcoming the limitations of sensitivity caused by Debye screening is expected to be a key issue in advancing cross-scale sensing, which aims to link molecular-level information with macroscopic environmental processes.

### Emerging technologies

#### Surface plasmon polariton-based optical sensing

To overcome the Debye screening limitation in high-ionic-strength environments, using functional layers with short-range molecular recognition mechanisms (e.g., aptamers) or employing transistor devices with nanoscale channel thickness are promising strategies. Alternatively, sensing modalities that detect signals beyond the Debye screening length are another promising way to overcome this limitation. One of the representative examples is surface plasmon polaritons (SPPs) [19]. SPPs are waves formed by the collective motion of electrons at a metal surface, which create a localized electromagnetic field that extends slightly into the surrounding medium. The evanescent electromagnetic field typically extends about 100 to 200 nm into the medium, depending on the wavelength of the incident light and the dielectric properties of the material. Molecular binding events at the metal surface can be detected by SPPs based on changes in the local refractive index, because the sensing field extends beyond the Debye screening length even in environments with high ionic strength. Recent advances in multimodal interfacial characterizations further emphasize the utility of SPR-based sensing platforms for evaluating molecular adsorption and binding kinetics at solid–liquid interfaces [20]. In particular, integrating functional bio-interfaces onto optical fiber-based SPR platforms has emerged as a practical approach for real-time monitoring of molecular interactions and adsorption dynamics at solid–liquid interfaces [19,21]. This architecture provides a compact and flexible optical sensing platform suitable for marine applications. Indeed, SPR-based platforms have demonstrated practical utility in oceanography; for instance, portable SPR devices have been deployed for direct, sensitive detection of hazardous proteins in raw seawater [19]. Integrating such biorecognition platforms into optical fiber configurations further offers a direct-immersion approach for real-time, *in situ* biochemical sensing.

#### Nanoscale transistors (Nano-FETs)

NanoFET-based sensing devices are characterized by nanoscale channels and interfacial structures [22–24]. The application of nanoFETs is one of the promising approaches for chemical sensing under Debye screening constraints as they efficiently transduce surface potential modulation into electrical signals. The key significance of nanoFETs lies in nanoscale structures that directly translate molecular-scale phenomena into electrical signals. Therefore, transistor technologies based on nanomaterials and nanoscale interfacial design become a crucial technological foundation for advancing cross-scale sensing under high-ionic-strength conditions, including marine environments. In underwater environments, some metal ions show toxic effects on ecosystems, while certain kinds of trace metals, such as iron, also function as essential factors for biological activity [25]. Therefore, the highly sensitive and quantitative determination of dissolved metal ions is important not only for assessing ecosystem impacts but also for understanding material cycling in the ocean and for exploring underwater mineral resources. As one of the examples, nanoFET-based sensors with selective membranes have been studied for the detection of heavy metal ions in aqueous environments [26].

To address Debye screening limitations, cross-scale target conversion strategies, wherein molecular recognition events are transformed into localized secondary chemical or ionic signals, offer an effective approach for high-salinity sensing. For example, Ono et al. integrated a graphene FET with femtoliter microfluidic droplets to detect pathogens captured by antibodies beyond the Debye screening length. In this system, a target-associated enzyme converts the substrate into diffusible ammonia, which accumulates in the microdroplet and directly adsorbs onto the graphene channel to yield an electrical signal [27]. Furthermore, adopting CRISPR-Cas enzymatic reactions serves as a powerful signal conversion strategy to indirectly detect target DNA that is otherwise challenging to capture directly in high-salinity environments. This approach effectively broadens the applicability of electrical and optical sensing devices for marine biosensing [28].

In recent years, a new approach has been proposed in which ion-specific interfacial interactions are directly converted into electrical signals using a nanoFET. Sivakumarasamy et al. demonstrated the detection of multiple ion species in serum at physiological ionic strength, without the use of selective membranes, by employing nanotransistor-based ion sensors [29]. Furthermore, our recent work demonstrated the quantitative detection of lanthanum (La^3+^), a representative rare-earth element, using a liquid-gated nanoFET without selective recognition layers [30]. This approach presents a sensing mechanism distinct from conventional Debye screening-limited sensing by exploiting differential interfacial interactions of ions (non-coulombic ion-specific interactions) at the nanoscale channel surface. The limitation of this approach is the need to implement the standard addition calibration method. However, it has the advantage of suppressing long-term drift as the calibration is inherently embedded within the measurement process. Furthermore, such calibration procedures can be integrated and automated using lab-on-a-chip (LOC) technology, making them effective for *in situ* environmental sensing.

#### Molecular recognition at the nanoscale

In cross-scale sensing, target molecules and ions exhibit diverse geometries, sizes, and charges; therefore, appropriate recognition materials should be designed on principles of molecular recognition chemistry. In this context, detection mechanisms can be classified into two types, namely, selective and cross-reactive detection. The specificity of recognition materials based on the lock-and-key principle enables selective detection through high binding affinity. Meanwhile, as observed in the mammalian olfactory system, simultaneous detection is achieved through combinatorial responses of multiple cross-reactive receptors exhibiting moderate binding affinities. For example, aptamers represent one of the possible recognition elements due to their small size, structural tunability, and compatibility with high-ionic-strength conditions; they have been applied to FET-based chemical sensors for selective detection [31]. Under Debye screening conditions, aptamers are practical recognition elements because their flexible three-dimensional structure allows bonding events and the resulting charge redistribution to occur in the vicinity of the semiconductor surface. Localized rearrangements of the structure of the molecular backbone can still induce detectable changes of the interfacial potential at the sensing surface, even when the molecular length exceeds the Debye length under physiological conditions [17,32]. Actually, notable results have been reported where organic FETs functionalized with DNA aptamer-modified gold nanoparticles successfully achieved selective target detection even in undiluted seawater [33].

As another example, enzyme-based reaction sensing mechanisms also represent a promising approach. Enzymatic reactions induce local changes in proton concentration and other ionic species at the sensor interface, enabling effective signal transduction via ISFETs even under Debye-screened conditions [34]. For applications in extreme environments, such as the deep sea, characterized by high pressure and low temperature, it is necessary to carefully assess possible changes in enzyme activity and stability prior to deployment of the sensor devices based on enzymatic reactions. As outlined above, nanoscale molecular design of recognition elements is essential not only for selectivity but also for effective electrical readout. The integration of molecular recognition mechanisms with transistors has utilized a variety of approaches for clinical applications [35].

In contrast to recognition materials based on the lock-and-key principle, molecular self-assembly systems have been investigated as effective recognition platforms for simultaneous detection in aqueous environments [36]. Such systems are attractive because they utilize reversible interactions to construct cross-reactive chemical sensors without requiring complex synthetic processes. For example, Sasaki et al. reported a molecular self-assembled chemical sensor array based on the reversible formation and substitution reactions of boronate esters between target molecules and building blocks of chemical sensors. Spontaneous association and dissociation governed by dynamic covalent bonds generate distinct response patterns, enabling the qualitative identification and quantitative estimation of multiple metal ions, even under competitive ion conditions, using machine learning methods [37]. Furthermore, the concept of molecular self-assembly was expanded to a coordination binding-based sensor array utilizing catechol dyes and zinc(II) ions for oxyanion detection [38]. This chemical sensor array achieved both qualitative identification and quantitative measurement in natural water samples without any sample treatment. Currently, these sensor devices are being actively developed as simple and disposable paper-based technologies for rapid on-site analysis [39]. To apply this approach to marine environments, translating these self-assembled sensing chemistries into LOC-based colorimetric device formats or optodes represents a promising strategy for multiparameter underwater sensing. A systematic overview of these field-proven and emerging cross-scale sensing technologies is summarized in Table 1.

**Table 1:** Overview of field-proven and emerging sensing technologies for cross-scale marine environmental monitoring.

Technology	Major target	Core sensing principle	Advantages	Current limitations	Ref.

**Field-proven technologies**					
pH-ISFETs	carbonate system parameters (pH, *p*CO_2_, total alkalinity)	gate potential change via surface protonation/deprotonation	high stability, fast response, high pressure tolerance, CMOS compatibility	reference electrode drift, biofouling, need for periodic calibration	[7–15]

**Emerging technologies**					
surface plasmon polaritons	biomolecules (e.g., hazardous proteins), dissolved gases	surface plasmon resonance at metal–dielectric interfaces	label-free detection, non-coulombic sensing	temperature and salinity cross-sensitivity, surface biofouling	[19–21]
nanoscale transistors	specific ions (e.g., trace/heavy metals), nucleic acids	charge sensitivity and non-coulombic ion interactions at nanoscale interfaces	ultrahigh sensitivity, high miniaturization, low power consumption	severe Debye screening in seawater, complex nanofabrication	[22–26,29–30]
molecular recognition at the nanoscale	small molecules, trace metals, biomarkers	conformational changes bringing charges within the Debye length, dynamic self-assembly and competitive ligand/ion displacement	can mitigate Debye screening limitations through conformational/charge-redistribution mechanisms	challenges in instrumentation and system integration, biofouling, receptor/enzyme instability	[17,31–39]

### Enabling technologies

#### Lab-on-a-chip technology for fluid handling

As discussed earlier, nanoscale transistor technologies, functionalized sensing interfaces, and molecular recognition strategies have expanded the scope of *in situ* chemical measurements. Notably, some of these technologies have demonstrated sensing capability even under high-ionic-strength conditions, suggesting their relevance for challenging environments such as marine environments. However, practical implementation of these novel sensing technologies in real-world environments needs strategic design of the entire measurement system, including fluid handling for sample and reagent delivery and pretreatment. Analytical assays including sample pretreatment, reagent mixing, and chemical reactions fundamentally require integration with precise fluid handling systems integrated on sensor devices. In addition, a flow system is often required to enable sensor calibration for drift compensation, which can become significant during long-term continuous operation. Integrated flow systems may also enable periodic cleaning procedures to mitigate biofouling of the sensing interface, thereby reducing sensor drift during long-term operation [12]. Moreover, the integration of flow systems with optical components is essential for analytical measurements based on molecular recognition strategies utilizing colorimetric principles, which require controlled mixing of samples and reagents. In label-free nanoFET sensor devices based on ion-specific interfacial interactions, a series of standard solutions, in principle, must be introduced quantitatively to obtain corresponding sensor responses. LOC technologies have been increasingly developed as an essential approach for implementing flow-based systems in field applications [40–41]. For example, LOC technology has been applied to continuous measurements [42–43] to achieve miniaturization of *in situ* spectrophotometric pH measurement systems. By integrating microfluidic channels, mixers, pumps, and valves, *in situ* calibration and automated measurement sequences become possible for long-term operation of chemical sensors.

In marine LOC devices, achieving efficient reagent mixing under high Péclet number (Pe) conditions without excessive pressure drop is a key design challenge. To prevent measurement errors caused by Taylor–Aris dispersion during *in situ* calibration, microfluidic handling must minimize boundary mixing between chemicals and samples or calibration standards. Droplet microfluidics is a powerful tool offering rapid internal convection. For optical or rapid colorimetric LOC systems, segmented microfluidics using droplet or gas-plug boundaries can effectively reduce axial dispersion [40]. However, oil-phase contamination may interfere with FET sensing and affect sensor performance. Single-phase continuous microfluidics with low-resistance passive mixing may therefore be advantageous for simple and robust *in situ* sensing devices. Passive mixing structures, such as curved microchannels inducing Dean flow or grooved channels generating chaotic advection [44], can promote mixing while maintaining relatively low hydrodynamic resistance. Furthermore, for long-term monitoring where rapid sample switching is not required, stop-flow operation [40] can be leveraged to enable passive diffusive mixing while reducing flow-induced dispersion and continuous pumping power consumption.

While LOC technologies enable the miniaturization of sensors and *in situ* analysis devices, the power consumption of pumping systems remains a major challenge for long-term deployment. It is a critical issue especially for the deployments using well-miniaturized ocean observation platforms with limited electric power supply. Furthermore, stable fluid flow with minimal pulsation is needed for nanoscale sensing particularly in freshwater or low-ionic-strength solutions because streaming potentials may influence the electrical response of nanoscale transistor sensors [45]. Therefore, the successful field deployment of nanoscale sensing technologies depends on the practical implementation of power-efficient and stable pumping technologies. In recent years, low-power or passive pumping technologies have been explored for LOC systems as alternatives to mechanically actuated pumps and valves, which typically require substantial electrical power [46]. Recent advances and practical integrations of power-efficient and power-free pumping technologies specifically targeted for *in situ* underwater measurements have also been discussed in a previous review [47]. One example of a stable and energy-efficient pumping technology is the electroosmotic pump (EOP), which is based on electroosmosis. By applying an electric field along a microchannel with charged walls, the motion of counterions within the electric double layer is induced. Through viscous coupling, this ionic motion results in bulk fluid flow [48]. One of the advantages of EOPs is the ability to generate a stable flow with minimal pulsation, owing to the absence of mechanically moving parts. However, in high-ionic-strength solutions, such as physiological fluids or seawater, the efficiency of EOPs may decrease due to increased electrical conductivity and Joule heating [48]. Although high conductivity can make the direct pumping of certain samples challenging, it has also motivated the development of advanced indirect pumping approaches that utilize working fluids to maintain efficiency. EOPs can also serve as effective pressure sources for actuating integrated microvalves and other fluidic components that operate intermittently during measurements. For example, the applicability of an *in situ* LOC chemical analyzer with EOPs as actuators for on-chip valves and a flow-rate regulator within a microfluidic device was shown through chemiluminescence-based quantitative determination of manganese in seawater [49].

A power-free pumping technology already in practical use in the medical field is the infusion balloon pump [50]. This medical device, driven by the elastic contraction of a balloon, is used for the continuous delivery of medicine that is filled into an expandable balloon to a patient. It offers the advantage of continuous flow through a simple structure without requiring an external power supply. This mechanism generates no pulsation because it relies on the steady contraction of the inflatable balloon. The practical applicability of balloon pumps has been demonstrated through their integration into LOC-based *in situ* biochemical analyzers. Their pumping performance remained stable even under elevated hydrostatic pressure of up to 20 MPa (corresponding to a depth of approximately 2,000 m), demonstrating their robustness for deep-sea applications [51]. Pumping mechanisms based on the expansion of elastomer membranes can also be integrated into LOC devices, providing a simple and power-free approach for fluid handling [52].

Osmotic pumps, generally used for continuous drug delivery in living animals, are another off-the-shelf power-free pumping solution. They rely on osmotic pressure across a semipermeable membrane to drive fluid flow and maintain a nearly constant drug release rate [53]. The Osmo pump is a small pump with no moving parts other than a semipermeable membrane, enabling long-term fluid delivery; however, its flow rate is relatively limited when used alone. Furthermore, it requires a long time for the flow rate stabilization during the initial stage of operation. This technology has already been applied to LOC devices for the quantitative analysis of metal ions [54] and for sampling [55] in deep-sea environments. These power-free pumping technologies may be well suited for housekeeping fluidic operations that support long-term observations, such as the continuous cleaning of sensor devices.

**Figure 2:**
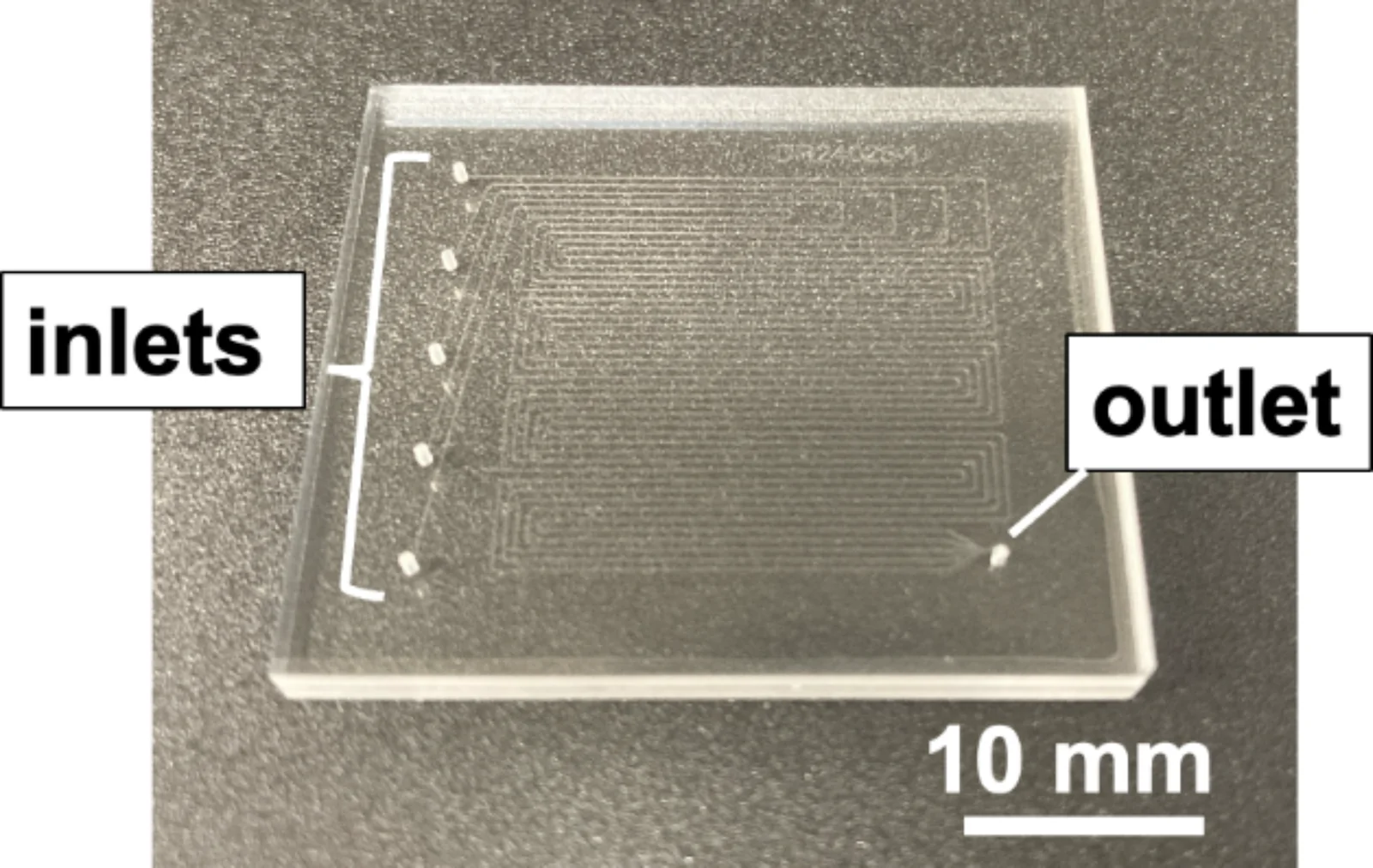
Microfluidic pressure reducer (MPR) with a branched microchannel.

Hydrostatic pressure rises by approximately 0.1 MPa for each 10 m of depth in aquatic environments. Therefore, in most cases, analytical instruments, electronics, and batteries deployed underwater must be placed in pressure-tight housings to protect them from hydrostatic pressure. In recent years, a method for pumping that utilizes the hydrostatic pressure difference between the inside and outside of a pressure-tight housing has been proposed [30,56–57]. By connecting the inside and outside of a pressure-tight housing via an appropriate flow resistance, such as a capillary tube, power-free pumping driven by hydrostatic pressure can be achieved. According to the Hagen–Poiseuille equation, the flow rate is determined by pressure difference, liquid viscosity, and capillary dimensions. In deep-sea environments, long or narrow capillary tubes are required to decrease large pressure differences, which makes miniaturization challenging. A compact and practical system can be realized by integrating microfluidic channels as a microfluidic pressure reducer (MPR). Power-free pumping using a glass-based MPR has demonstrated stable delivery of both pure water and natural seawater up to 10 MPa of hydrostatic pressure [57]. Furthermore, a mechanism enabling stepwise flow-rate adjustments using MPR devices with branched microchannels has been proposed and is currently under evaluation (Figure 2). By combining this power-free pumping technology with an indirect pumping platform, practical fluid control operations can be realized. For example, integrating a 3D-printed fluidic platform designed for indirect pumping with a pH-ISFET enables the delivery of multiple pH standard solutions, and *in situ* calibration capability has also been demonstrated [56]. As discussed above, the integration of LOC technologies, power-free or power-efficient pumping strategies, and nanoscale sensors enables the deployment of advanced analytical methodologies in harsh field environments.

#### Extended-gate FET (EG-FET) technology

Considering the high ionic strength and harsh physicochemical conditions of marine environments, the development of appropriate recognition materials significantly expands the potential of chemical sensor platforms as discussed in the previous section. In addition to molecular design, device-level engineering is equally critical for the practical deployment of nanoscale sensing technology in marine environments. To date, ISFETs have shown effectiveness in chemical sensing under marine environments; however, robust device architectures are required to ensure operational stability and practical deployment in such extreme environments. The extended-gate FET (EG-FET) architecture is particularly well suited for implementing surface functionalization with molecular recognition layers, including enzymes [34]. The EG-FET configuration is designed to physically separate the transistor from the sensing element, allowing only the functionalized sensing surface to be exposed to the surrounding environments. This separation allows the transistor circuitry to be housed and protected within a pressure-tight housing while maintaining direct environmental interaction of the sensing surface. As a result, the configuration improves operational stability and protects the FET circuitry under high pressure and corrosive conditions, while also facilitating easy replacement of the sensing element. With the growing demand for mineral resource exploration and associated environmental impact assessment missions, sensing performed at depths of thousands of meters is becoming increasingly important. While such conditions remain largely unexplored for Nano-FET technologies, significant changes in operating mechanisms are expected under deep-sea conditions, which must be studied in the future.

Deploying thin-film NanoFETs with microfluidics in deep-sea environments presents material challenges, particularly interfacial delamination under shear stress and high pressure. To improve layer stability and prevent pinhole leakage, possible fabrication strategies include combining high-density deposition methods (e.g., atomic layer deposition or sputtering) with adhesion layers, such as Ti or Cr [8,15]. Post-deposition thermal annealing may also help relieve internal stress and densify the dielectric matrix, depending on the materials and fabrication process [8].

Connecting nanoscale sensors to macroscale fluidic packaging requires a hierarchical fabrication approach to prevent dead volume and sample dispersion. While 3D printing provides robust macroscale manifolds, its spatial resolution cannot interface directly with nanochannels at present. Therefore, intermediate microfluidic interfaces can be fabricated using photolithography and soft lithography with polydimethylsiloxane (PDMS) to form micrometer-scale channels [58]. Elastomeric PDMS serves a dual purpose by functioning as a flow cell and acting as a gasket that seals rigid sensor chips against 3D-printed housings, which may help reduce pressure-induced mechanical stress and fluid leakage.

#### Observation platform technology

To deploy sensor devices in environments such as the ocean, appropriate mounting platforms are indispensable. In particular, for spatiotemporally resolved deployments aimed at cross-scale sensing, the use of scalable platform technologies is essential. In recent years, uncrewed observation platforms have gained increasing importance as alternatives to conventional ocean observation systems based on crewed research vessels [59]. This section provides an overview of platform technologies that link nanoscale sensing techniques and global environmental monitoring.

A moored observatory is one of the most fundamental platforms for long-term, continuous observation of the marine environment at fixed locations. By deploying sensor devices from the surface to the seafloor, long-term observation of biogeochemical parameters with high temporal resolution becomes possible. Installation and retrieval of observation buoys require costly shipboard operations, sensor calibration, maintenance, and biofouling prevention measures. Therefore, to achieve long-term and sustainable observations, it is important to design cost-effective observation systems and establish effective operational procedures [60]. In the future, more efficient observation systems will need to be developed by miniaturizing moored buoys and introducing technologies that enable automated deployment, recovery, maintenance, and sensor replacement, thereby supporting sustained ocean observations.

**Figure 3:**
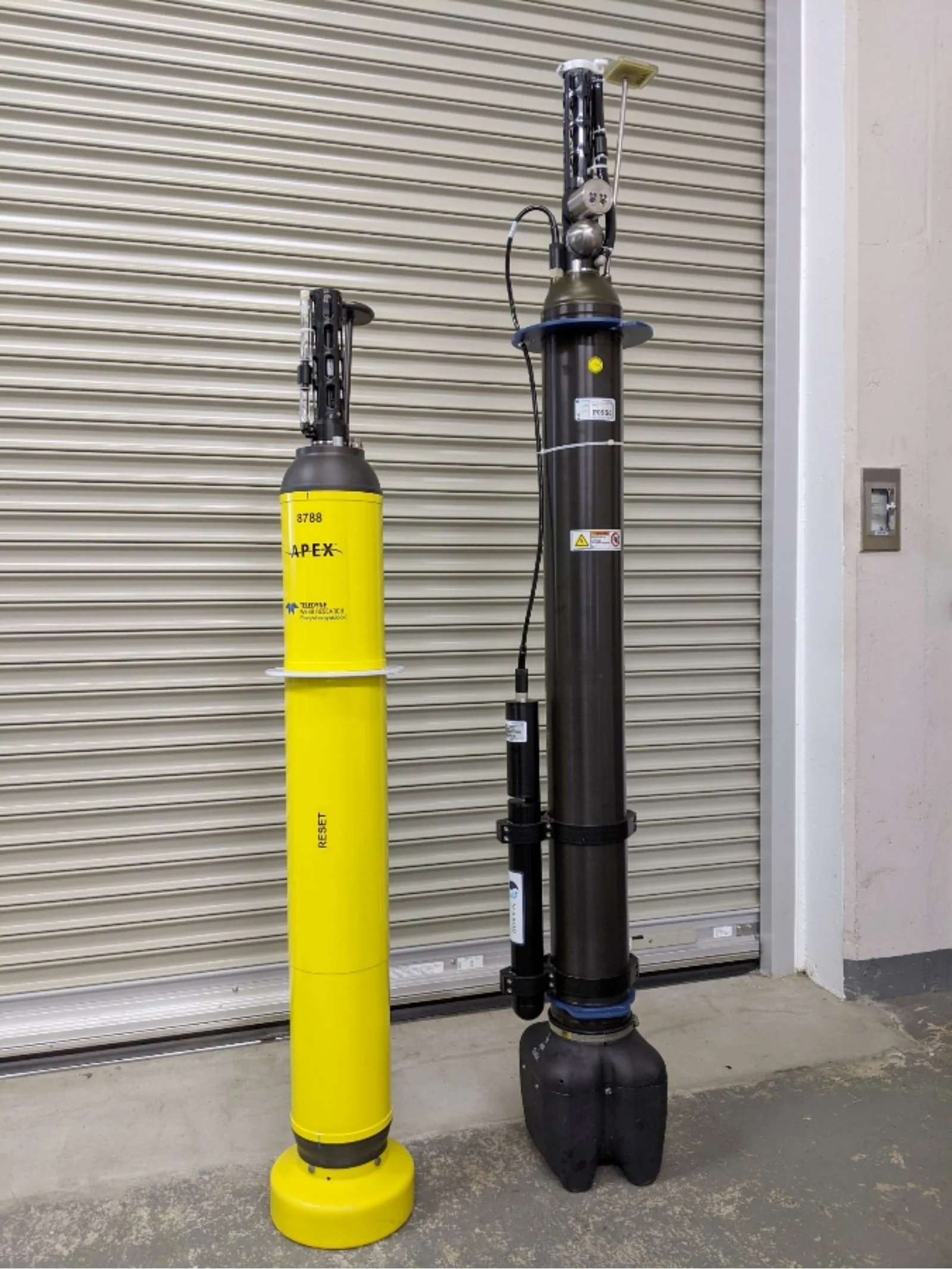
Argo float (left) and BGC Argo float (right), © JAMSTEC. Reproduced with permission. This content is not subject to CC BY 4.0.

Another approach is the use of virtual mooring technology based on various autonomous surface vehicles (ASVs) or autonomous underwater gliders [61]. Deploying a miniaturized virtual mooring platform using an aircraft or other means could enable timely deployment of observation tools and is expected to become a promising approach for future ocean observation [62–63]. In terms of power supply, a certain amount of electricity can be generated using solar panels mounted on a surface float. Furthermore, some ocean platforms can harvest multiple forms of renewable energy, including wind, waves, and ocean currents. For example, some unmoored platforms integrate wave-induced turbines or subsurface wave energy harvesters to achieve long-term, self-sustained operations [62]. Drifting buoys, especially profiling floats, are representative examples of the successful realization of a global ocean observation network. Thousands of autonomous profiling floats are deployed throughout the oceans to continuously observe basic physical parameters such as water temperature and salinity [2]. Recent expansions through the BGC-Argo program have enabled observations of additional parameters [3] (Figure 3). These profiling floats are subject to strict constraints on size, power consumption, and payload weight; therefore, the integration of small, low-power sensing devices is essential [64]. In recent years, deployment of profiling floats in coastal areas has also been reported. While traditional drifting buoys can lead to unexpected operational conflicts in coastal environments due to their unpredictable trajectories, certain advanced floats have been studied and developed to minimize these risks by temporarily anchoring to the seabed [65]. However, the operational scope of these drifting platforms remains constrained by their reliance on environmental conditions or specific seabed characteristics. To overcome these limitations, platforms capable of more flexible movement underwater and on the sea surface will become increasingly important. In recent years, compact remotely operated vehicles [66], autonomous underwater vehicles [67], underwater gliders [61], and ASVs [68] have become available off-the-shelf for practical missions.

For the rapid transport and deployment of sensor devices to remote places, unmanned aerial vehicles are also useful ocean observation platforms [69]. They also support simultaneous optical sensing using onboard imaging sensors [59], allowing *in situ* measurements to be complemented with high-resolution spatial observations of the surrounding marine environment. However, there is a bottleneck on payload weight and power capacity. Therefore, further miniaturization and reduced power consumption of sensor devices are essential. While *in situ* sensing platforms provide detailed local measurements, airborne and satellite remote sensing technology play an important complementary role by enabling observations across broader spatial scales. Active optical sensing technologies, such as lidar, can also be used to estimate biogeochemical parameters of water columns based on laser backscatter signals [70]. These approaches emphasize the potential of remote sensing to capture large-scale environmental variability. In contrast, *in situ* chemical sensing technologies provide direct measurements of dissolved chemical species and molecular-scale processes in aquatic environments. The integration of *in situ* nanoscale sensing and remote sensing offers a complementary strategy for linking molecular-level processes with global environmental dynamics across scales.

## Future Directions

**Figure 4:**
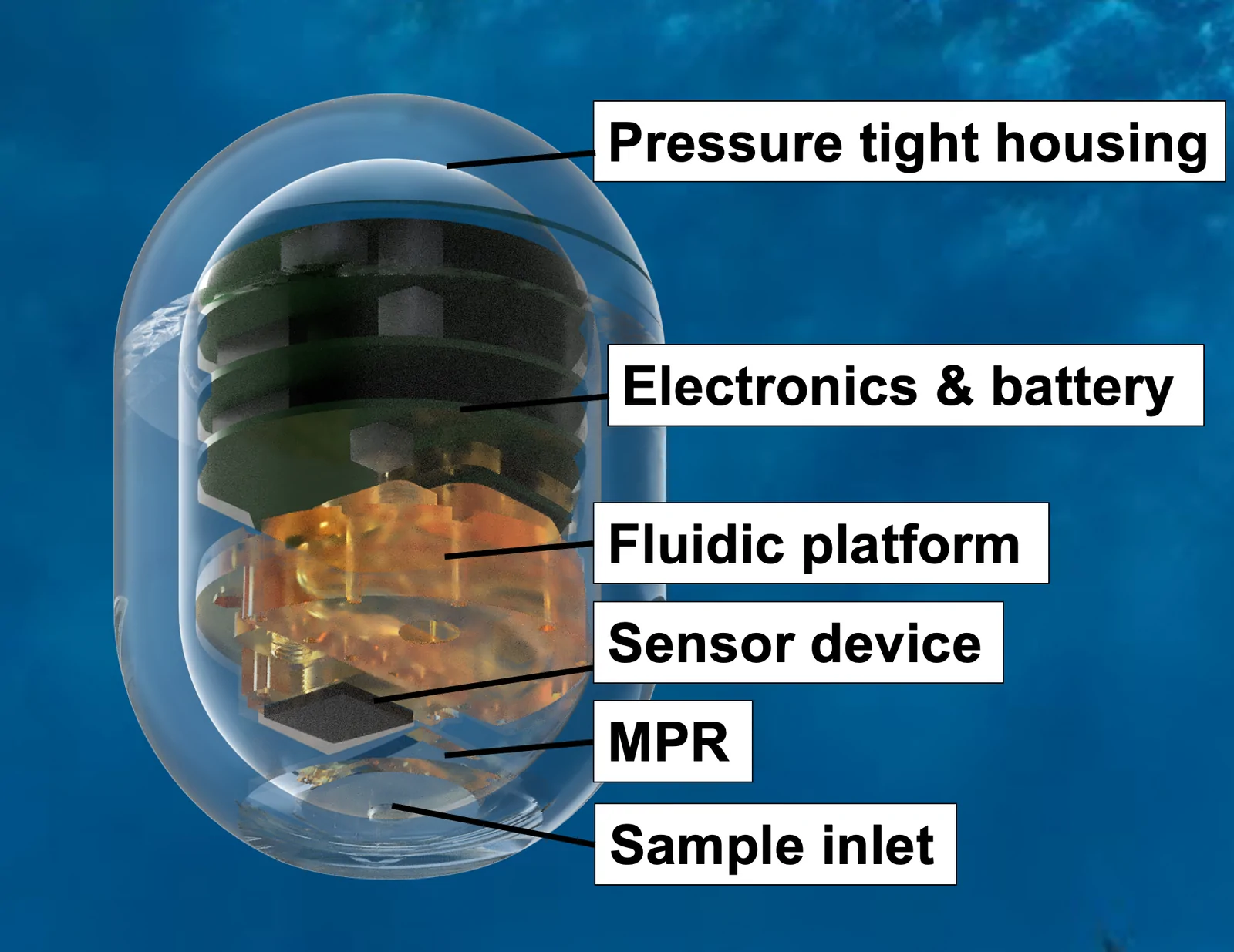
Concept of compact and field-deployable sensing platform.

As outlined here, nanoscale sensing technologies are steadily expanding the potential for sensing in high-ionic-strength environments, such as oceans and biological systems. The pH-ISFET, already in practical use for pH measurement in aquatic environments, can be regarded as a pioneering success story that links nanoscale interfacial phenomena to macroscopic oceanic observations, and ultimately, to global environmental issues. However, the direct electrical detection of specific targets using FET-based sensors is constrained by Debye screening, which continues to pose a fundamental challenge. The integration of nanoscale transistors, molecular recognition technologies, extended-gate structures, and power-efficient pumping technologies, as outlined here, offers a pathway towards underwater *in situ* sensing technologies while addressing these limitations. For example, the convergence of LOC technology and 3D printing enables the incorporation of power-efficient pumping systems, indirect pumping architectures, and protective packaging (pressure-tight housing) functionalities into compact, field-deployable sensing platforms (Figure 4). Despite promising technological advances, several fundamental limitations remain to be addressed before cross-scale marine sensing becomes fully operational. Debye screening in high-salinity seawater still restricts direct electrical detection of larger biomolecules or non-proton target ions, which motivates the use of short conformational receptors (e.g., aptamers) or non-coulombic sensing mechanisms. Potential drift in solid-state and liquid-junction reference electrodes over multimonth deployments remains an important challenge, highlighting the need for practical *in situ* calibration strategies. Surface biofouling impairs gate sensitivity and clogs microchannels; approaches such as non-fouling surface chemistry [71], deep-UV irradiation [72], or periodic microfluidic washing [12] may help mitigate these effects.

Both marine and physiological environments share a common physical constraint, that is, high ionic strength. On this shared foundation, nano-interface sensing technology has the potential to become a cross-disciplinary, cross-scale sensing platform for both fields. In the future, further miniaturization and higher levels of packaging, along with increasingly stringent demands for safety and ultralow-power operation, may be necessary to enable the application of nanoscale sensing technologies in continuous in vivo monitoring. Consequently, in addition to LOC technologies, pumping methods that operate with minimal or no power consumption are expected to become increasingly important. Historically, “bioanalytical-originated” devices such as ISFETs have been applied in marine environmental sensing. In contrast, “ocean-originated” miniaturized sensing technologies integrating nanoscale sensing and LOC technologies are expected to evolve into next-generation platforms in biomedical and healthcare fields, enabling bidirectional cross-disciplinary innovation in the future.

## Data Availability

Data sharing is not applicable as no new data was generated or analyzed in this study.

## References

[1] Doney S C, Fabry V J, Feely R A, Kleypas J A (2009). Annu Rev Mar Sci.

[2] Wong A P S, Wijffels S E, Riser S C, Pouliquen S, Hosoda S, Roemmich D, Gilson J, Johnson G C, Martini K, Murphy D J (2020). Front Mar Sci.

[3] Roemmich D, Alford M H, Claustre H, Johnson K, King B, Moum J, Oke P, Owens W B, Pouliquen S, Purkey S (2019). Front Mar Sci.

[4] Liu Y, Lu H, Cui Y (2023). J Mar Sci Eng.

[5] Dickson A G, Sabine C L, Christian J R (2007). Guide to Best Practices for Ocean CO2 Measurements.

[6] Seiter J C, DeGrandpre M D (2001). Talanta.

[7] Bergveld P (2003). Sens Actuators, B.

[8] Sinha S, Pal T A (2022). Electrochem Sci Adv.

[9] Le Bris N, Birot D (1997). Anal Chim Acta.

[10] Shitashima K, Maeda Y, Koike Y, Ohsumi T (2008). Int J Greenhouse Gas Control.

[11] Jimenez-Jorquera C, Orozco J, Baldi A (2010). Sensors.

[12] Bresnahan P J, Martz T R, Takeshita Y, Johnson K S, LaShomb M (2014). Methods Oceanogr.

[13] Johnson K S, Jannasch H W, Coletti L J, Elrod V A, Martz T R, Takeshita Y, Carlson R J, Connery J G (2016). Anal Chem (Washington, DC, U S).

[14] Hemming M P, Kaiser J, Heywood K J, Bakker D C E, Boutin J, Shitashima K, Lee G, Legge O, Onken R (2017). Ocean Sci.

[15] Briggs E M, Sandoval S, Erten A, Takeshita Y, Kummel A C, Martz T R (2017). ACS Sens.

[16] Stern E, Wagner R, Sigworth F J, Breaker R, Fahmy T M, Reed M A (2007). Nano Lett.

[17] Nakatsuka N, Yang K-A, Abendroth J M, Cheung K M, Xu X, Yang H, Zhao C, Zhu B, Rim Y S, Yang Y (2018). Science.

[18] Langmuir D (1997). Aqueous Environmental Geochemistry.

[19] Homola J (2008). Chem Rev.

[20] Yamada S, Urano H, Toma K (2026). Beilstein J Nanotechnol.

[21] Kitaya N, Yamanaka D, Morita K, Naito R, Suhara H, Suzuki K, Nakajima H, Moriiwa Y, Morioka K, Yanagida A (2026). Beilstein J Nanotechnol.

[22] Cui Y, Wei Q, Park H, Lieber C M (2001). Science.

[23] Ohno Y, Maehashi K, Yamashiro Y, Matsumoto K (2009). Nano Lett.

[24] Sarkar D, Liu W, Xie X, Anselmo A C, Mitragotri S, Banerjee K (2014). ACS Nano.

[25] Morel F M M, Price N M (2003). Science.

[26] Liu C, Ye Z, Wei X, Mao S (2022). Electrochem Sci Adv.

[27] Ono T, Kanai Y, Inoue K, Watanabe Y, Nakakita S-i, Kawahara T, Suzuki Y, Matsumoto K (2019). Nano Lett.

[28] Kim N, Collins D S, Donghia N M, Miller B S, Sallum H M, Lybbert S R, Perini E, Niemi J B, Collins J J, Nguyen P Q (2026). Nat Sustainability.

[29] Sivakumarasamy R, Hartkamp R, Siboulet B, Dufrêche J-F, Nishiguchi K, Fujiwara A, Clément N (2018). Nat Mater.

[30] Fukuba T, Fujiwara A, Nishiguchi K, Bergaud M, Grall S, Li S, Kim S-H, Clément N (2023). Application of Ambient Pressure-Driven Pumping Technology Towards Ultra Low-Power Underwater Sensing. 2023 IEEE Underwater Technology (UT).

[31] Song S, Wang L, Li J, Fan C, Zhao J (2008). TrAC, Trends Anal Chem.

[32] Maehashi K, Katsura T, Kerman K, Takamura Y, Matsumoto K, Tamiya E (2007). Anal Chem (Washington, DC, U S).

[33] Knopfmacher O, Hammock M L, Appleton A L, Schwartz G, Mei J, Lei T, Pei J, Bao Z (2014). Nat Commun.

[34] Sarcina L, Macchia E, Tricase A, Scandurra C, Imbriano A, Torricelli F, Cioffi N, Torsi L, Bollella P (2023). Electrochem Sci Adv.

[35] Poghossian A, Schöning M J (2014). Electroanalysis.

[36] Sasaki Y, Kubota R, Minami T (2021). Coord Chem Rev.

[37] Sasaki Y, Minamiki T, Tokito S, Minami T (2017). Chem Commun.

[38] Hamedpour V, Sasaki Y, Zhang Z, Kubota R, Minami T (2019). Anal Chem (Washington, DC, U S).

[39] Sasaki Y, Minami T (2026). Beilstein J Nanotechnol.

[40] Nightingale A M, Beaton A D, Mowlem M C (2015). Sens Actuators, B.

[41] Fukuba T, Fujii T (2021). Lab Chip.

[42] Rérolle V M C, Floquet C F A, Mowlem M C, Connelly D P, Achterberg E P, Bellerby R R G J (2012). TrAC, Trends Anal Chem.

[43] Yin T, Papadimitriou S, Rérolle V M C, Arundell M, Cardwell C L, Walk J, Palmer M R, Fowell S E, Schaap A, Mowlem M C (2021). Environ Sci Technol.

[44] Suh Y K, Kang S (2010). Micromachines.

[45] Newaz A K M, Markov D A, Prasai D, Bolotin K I (2012). Nano Lett.

[46] Luo X, Yang L, Cui Y (2023). Sens Actuators, A.

[47] Fukuba T (2025). Bunseki Kagaku.

[48] Li L, Wang X, Pu Q, Liu S (2019). Anal Chim Acta.

[49] Provin C, Fukuba T, Okamura K, Fujii T (2013). IEEE J Oceanic Eng.

[50] Skryabina E A, Dunn T S (2006). Am J Health-Syst Pharm.

[51] Fukuba T, Nakasa A, Tsukada O, Fujii T (2018). Application of Drug Infusion Balloons to Microfluidic Systems for In Situ Measurement. Proceedings of the 22nd International Conference on Miniaturized Systems for Chemistry and Life Sciences (µTAS 2018).

[52] Morimoto Y, Mukouyama Y, Habasaki S, Takeuchi S (2016). Micromachines.

[53] Herrlich S, Spieth S, Messner S, Zengerle R (2012). Adv Drug Delivery Rev.

[54] Chapin T P, Jannasch H W, Johnson K S (2002). Anal Chim Acta.

[55] Jannasch H W, Wheat C G, Plant J N, Kastner M, Stakes D S (2004). Limnol Oceanogr: Methods.

[56] Fukuba T (2025). Application of Ambient Pressure-Driven Pumping Technology for Underwater pH Measurement. 2025 IEEE Underwater Technology (UT).

[57] Fukuba T (2024). Development of Power-Free Pumping Technology Utilizing Water Pressure in the Marine Environment. Proceedings of the 41st Sensor Symposium on Sensors, Micromachines and Applied Systems.

[58] Duffy D C, McDonald J C, Schueller O J A, Whitesides G M (1998). Anal Chem (Washington, DC, U S).

[59] Yuan S, Li Y, Bao F, Xu H, Yang Y, Yan Q, Zhong S, Yin H, Xu J, Huang Z (2023). Sci Total Environ.

[60] Venkatesan R, Ramesh K, Kishor A, Vedachalam N, Atmanand M A (2018). Front Mar Sci.

[61] Petritoli E, Leccese F (2025). Drones.

[62] Ticona Rollano F, Hume D, Martinez J (2021). Powering the Blue Economy: A Survey of Station-Keeping Methods for Mooringless Platforms.

[63] Wu D, Zhou R, Xia Q, Zhou P, Zhang X, Jin Z, Zhang Y, Yang C (2025). Ocean Eng.

[64] Johnson K S, Coletti L J, Jannasch H W, Sakamoto C M, Swift D D, Riser S C (2013). J Atmos Oceanic Technol.

[65] Andre X, Dutreuil V, Le Reste S (2016). Sea Technol.

[66] Singh R, Sarkar P, Goswami V, Yadav R (2022). Ocean Eng.

[67] Pairaud I, Petton S, Sellet H, Helleringer C, Le Bihan C, Charria G (2025). Using a micro-AUV for monitoring coastal river plume areas. Proc. IEEE OCEANS 2025 Brest.

[68] Bolbot V, Sandru A, Saarniniemi T, Puolakka O, Kujala P, Valdez Banda O A (2023). J Mar Sci Eng.

[69] Burnett M, Abdelwahab M, Satme J N, Downey A R J, Barahona Smith G, Fonce A, Imran J (2026). Sensors.

[70] Chen W, Chen P, Zhang H, He Y, Tang J, Wu S (2023). Intell Mar Technol Syst.

[71] Gokaltun A, Yarmush M L, Asatekin A, Usta O B (2017). Technology.

[72] Bueley C, Olender D, Bocking B (2014). J Ocean Technol.

